# Peroxidase (POD) Mimicking Activity of Different Types of Poly(ethyleneimine)-Mediated Prussian Blue Nanoparticles

**DOI:** 10.3390/nano15010041

**Published:** 2024-12-29

**Authors:** Udara Bimendra Gunatilake, Briza Pérez-López, Maria Urpi, Judit Prat-Trunas, Gerard Carrera-Cardona, Gautier Félix, Saad Sene, Mickaël Beaudhuin, Jean-Charles Dupin, Joachim Allouche, Yannick Guari, Joulia Larionova, Eva Baldrich

**Affiliations:** 1 ICGM, University of Montpellier, CNRS, ENSCM, 34000 Montpellier, France; gautier.felix@umontpellier.fr (G.F.); saad.sene@umontpellier.fr (S.S.); mickael.beaudhuin@umontpellier.fr (M.B.); yannick.guari@umontpellier.fr (Y.G.); joulia.larionova@umontpellier.fr (J.L.); 2 Diagnostic Nanotools Group, Hospital Vall d’Hebron Institut de Recerca (VHIR), 08035 Barcelona, Spain; briza.perez@vhir.org (B.P.-L.); maria.urpi@vhir.org (M.U.); judit.prat@vhir.org (J.P.-T.); gerard.carrera@alumni.vhir.org (G.C.-C.); 3 Universitat Autonoma de Barcelona (UAB), 08193 Bellaterra, Spain; 4 Institut des Sciences Analytiques et de Physicochimie Pour l’Environnement et les Matériaux, UMR 5254, E2S UPPA, CNRS, IPREM, 64000 Pau, France; jean-charles.dupin@univ-pau.fr (J.-C.D.); joachim.allouche@univ-pau.fr (J.A.); 5 Centro de Investigación Biomédica en Red de Enfermedades Infecciosas (CIBERINFEC), Instituto de Salud Carlos III, 28029 Madrid, Spain

**Keywords:** Prussian blue nanoparticles (PBNPs), nanozyme, poly(ethyleneimine) (PEI), peroxidase mimetic, amino-rich polymers, catalysts, catalytic inhibition, cyano-bridged coordination polymers

## Abstract

Prussian blue nanoparticles (PBNPs) have been identified as a promising candidate for biomimetic peroxidase (POD)-like activity, specifically due to the metal centres (Fe^3+^/Fe^2+^) of Prussian blue (PB), which have the potential to function as catalytically active centres. The decoration of PBNPs with desired functional polymers (such as amino- or carboxylate-based) primarily facilitates the subsequent linkage of biomolecules to the nanoparticles for their use in biosensor applications. Thus, the elucidation of the catalytic POD mimicry of these systems is of significant scientific interest but has not been investigated in depth yet. In this report, we studied a series of poly(ethyleneimine) (PEI)-mediated PBNPs (PB/PEI NPs) prepared using various synthesis protocols. The resulting range of particles with varying size (~19–92 nm) and shape combinations were characterised in order to gain insights into their physicochemical properties. The POD-like nanozyme activity of these nanoparticles was then investigated by utilising a 3,3′,5,5′-tetramethylbenzidine (TMB)/H_2_O_2_ system, with the catalytic performance of the natural enzyme horseradish peroxidase (HRP) serving as a point of comparison. It was shown that most PB/PEI NPs displayed higher catalytic activity than the PBNPs, with higher activity observed in particles of smaller size, higher Fe content, and higher Fe^2+^/Fe^3+^ ratio. Furthermore, the nanoparticles demonstrated enhanced chemical stability in the presence of acid, sodium azide, or high concentrations of H_2_O_2_ when compared to HRP, confirming the viability of PB/PEI NPs as a promising nanozymatic material. This study disseminates fundamental knowledge on PB/PEI NPs and their POD-like activities, which will facilitate the selection of an appropriate particle type for future biosensor applications.

## 1. Introduction

Biosensors typically employ the natural enzyme HRP to specifically detect target molecules. The enzyme’s catalytic POD activity, combined with a signal generator, converts the biochemical interaction into a measurable signal [1,2]. Nevertheless, natural enzymes present several limitations as a catalyst including low thermal and environmental stability, sensitivity to environmental conditions, non-conductivity, and the difficulty and expense of synthesis and purification, which has led to the utilisation of nanozymes [3,4]. In this regard, various types of artificial enzymes have been introduced including organic materials (for instance, fluorescein/quinone derivatives), metal organic framework-based materials (like Cu^2+^–bipyridine complexes), metal-based materials (such as Au, Pt, and Mo), and particularly Fe-based materials [5,6,7,8].

PB, (Fe(III)_4_[Fe(II)(CN)_6_]_3_), is one of the oldest discovered coordination polymer materials, consisting of ferric and ferrocyanide ions with a face centred cubic unit cell, where the nitrogen coordinates to Fe^3+^ and carbon coordinates to Fe^2+^. The physicochemical properties of PB including sorption, photo-magnetism, host–guest interactions, redox behaviour, and intrinsic porosity have been observed to result in a wide range of reported applications. It is noteworthy that PB materials have been approved by the U.S. Food and Drug Administration (FDA) for use as an antidote for humans in the event of poisoning by Cs^+^ and Tl^+^. Furthermore, PBNPs are employed in biomedical applications as an effective photothermal agent for photothermal therapy in cancer cells. They have also been utilised in gas capture and storage, Li^+^/Na^+^ batteries, sensors, theragnostic, and in nanoprobes for magnetic resonance imaging [9,10,11].

PB has been demonstrated to possess excellent optical and electrochemical properties, which have led to its exploitation in a variety of catalytic-based applications [12]. In this context, the Fe core is capable of acting as a catalytic centre due to its ability to electrochemically switch between multiple oxidation states including the fully oxidised, partially oxidised, and reduced forms of PB, which are Prussian yellow, Berlin green, and Prussian white, respectively [13,14]. Due to their strong electron transport ability, PBNPs are capable of mimicking the multienzyme-like activity exhibited by POD, catalase (CAT), and superoxide dismutase (SOD), indicating it as a promising enzymatic mimetic inorganic material [15].

In biosensor applications that detect analytes by linking them to enzymes via biomolecules (such as antibodies), thus generating a chromatic signal, the interaction between the nanozyme and these biomolecules is of great importance [16,17]. Therefore, to enhance biomolecule binding, nanozymes can be decorated with aminated polymers, such as PEI, which is prompting a growing need to investigate the POD-mimicking activity of these modified nanoconstructs. In this regard, Liang et al. synthesised PEI coated cubic shape PBNPs with a 50 nm edge length by the one-pot thermal reduction protocol [18]. These particles were reported as a pH switchable nanozyme displaying alternatively POD-like and CAT-like catalytic properties while shifting the pH from acidic to basic, respectively. Interestingly, the highest POD-like activity was observed at pH 5. Using a different approach, Pandey et al. synthesised PEI mediated copper-iron hexacyanoferrate (Cu/Fe HCFs) and nickel-iron hexacyanoferrate (Ni/Fe HCFs) nanoparticles by reducing potassium ferricyanide by PEI at 60 °C [19]. They optimised the metal ratios, finding that a 1:1 Cu:Fe molar ratio and a 1:5 Ni:Fe molar ratio resulted in nanoparticles with electrochemical properties similar to PB. Additionally, both Cu/Fe HCFs and Ni/Fe HCFs displayed POD-like activity, with Cu/Fe HCFs exhibiting a higher catalytic performance compared to Ni/Fe HCFs. Furthermore, only a few studies have described the use of electrodes modified with PB and PEI layers. Pchelintsev et al. reported PEI/PB coated screen printed electrodes, subsequently modified with glucose oxidase (GOx), as an amperometric biosensor for glucose [20]. Pajor-Świerzy et al. employed PEI to anchor PB/poly(allylamine hydrochloride) (PAH) multilayers on gold electrodes, thereby enhancing the electrochemical properties of the film through the addition of a conductive polymer, poly(3,4-ethylenedioxythiophene)–poly(styrenesulphonate) (PEDOT:PSS). In this case, the incorporation of the conductive polymer into the multilayers resulted in an improvement in the electrocatalytic sensitivity of hydrogen peroxide (H_2_O_2_) detection [21]. Additionally, PB/PEI–graphene multilayer films were reported to exhibit excellent electrochromic properties and good electrocatalysis towards H_2_O_2_ [22].

The majority of the PB/PEI NPs employed in nanozymatic studies have been synthesised through either the thermal reduction of ferricyanides in the presence of ferric salts and PEI, or with co-precipitation techniques in the presence of PEI (in situ/ex situ), with ferrocyanide/ferric salt injection. Nevertheless, little is known about the influence of the fundamental characteristics of PB/PEI NPs, which are derived from the synthesis technique, on the nanozymatic-POD-like activity. In this study, three distinct protocols were employed to synthesise a variety of PB/PEI NPs. The first protocol involved a controlled co-precipitation approach, whereby the controlled in situ synthesis of PBNPs was conducted within a PEI bath. The second protocol entailed a thermal reduction method, wherein the reduction of ferricyanide was achieved in the presence of PEI and ferric salt. Finally, the third protocol involved a vortex synthesis approach. For the latter, the PB/PEI NPs were precipitated within a custom-built vortex reactor. To the best of our knowledge, this is the first time that PB/PEI NPs synthesised by protocols (1) and (3) have been reported. The particles were characterised by transmission electron microscopy (TEM), X-ray diffraction (XRD), ultraviolet–visible spectroscopy (UV–Vis), X-ray photoelectron spectroscopy (XPS), Fourier transform infrared spectroscopy (FTIR), scanning electron microscopy (SEM) coupled with energy dispersive X-ray spectroscopy (EDX), and cyclic voltammetry (CV), and their nanozymatic-POD-like activity was investigated in order to elucidate the influence of the physical, chemical, and electrochemical properties of the particles, which were derived from the synthesis protocol, on their POD-like activity.

## 2. Experimental

### 2.1. In Situ Synthesis of PB/PEI Nanoparticles by Controlled Coprecipitation (**C1**)

All synthesis protocols were conducted in accordance with the relevant safety precautions, specifically within a ventilated area or within a fume hood. The first type of PB/PEI NPs was synthesised by coprecipitating PB inside a PEI bath. First, 0.5 g of branched PEI (M_w_~25,000, Sigma Aldrich, Steinheim, Germany) was dissolved in 100 mL of 0.25 M hydrochloric acid (HCl; Sigma Aldrich, ≥37.0%). Subsequently, 40 mL of 10 mM FeCl_3_.6H_2_O (Sigma Aldrich, 97%) and 40 mL of 11.25 mM Na_4_Fe(CN)_6_.10H_2_O (Acros Organics, Geel, Belgium) were injected into the 0.5% PEI solution at the rate of 4 mL/h (utilising a peristaltic pump) under 400 rpm stirring for a period of 10 h. The reaction temperature was maintained at 25 °C with the assistance of a water chiller. After completion of the reaction, the particles were separated by centrifugation (15,000 rpm, 15 min), after which they were washed with pure water 3 times and with ethanol (Carlo Erba, Val-de-Reuil, France, 96%) once. The purified particles (**C1**) were stored in pure water for further investigation. Mass obtained = 70 mg.

The same protocol was then followed to synthesise bare PBNPs (**Cb**) by injecting the same molar ratio and volumes of FeCl_3_.6H_2_O and Na_4_Fe(CN)_6_.10H_2_O into a 100 mL pure water bath, with the aforementioned conditions remaining fixed. Mass obtained = 80 mg, yield = 93%.

### 2.2. Synthesis of PB/PEI Nanoparticles by Thermally Reductive Process

Three types of PB/PEI NPs were synthesised through a thermally reductive process by modifying a previously reported protocol [23]. All precursors and PEI solutions were prepared in 0.1 M HCl (e.g., the 3% (*W*:*V*) PEI solutions used were prepared by dissolving 3 g of PEI in 100 mL of 0.1 M HCl).

#### 2.2.1. **T1**—PB/PEI Nanoparticles

First, 10 mL of 20 mM of FeCl_3_.6H_2_O, 10 mL of 22.5 mM K_3_Fe(CN)_6_ (Alfa Aesar, Haverhill, MA, USA; 99%), and 3% (*W*:*V*) PEI solutions were prepared by dissolving the salts and the polymer in 0.1 M HCl solution, respectively. Then, 10 mL of 22.5 mM K_3_Fe(CN)_6_ and 2 mL of 3% PEI were mixed, and 10 mL of the FeCl_3_.6H_2_O solution was injected at a rate of 20 mL/h for 30 min while stirring the mixture at 400 rpm at room temperature. The resultant solution was transferred to a 100 mL double neck round bottle flask, was vigorously stirred, heated up to the refluxing temperature, and was then refluxed for 30 min. After the sample temperature dropped to around 30 °C, the nanoparticles were separated by centrifugation (20,000 rpm, 15 min). Finally, the particles were washed with pure water twice and with ethanol once, and were stored in pure water for further investigation. Mass obtained = 50 mg.

#### 2.2.2. **T2**—PB/PEI Nanoparticles

A total of 10 mL of 10 mM of FeCl_3_.6H_2_O, 10 mL of 11.25 mM K_3_Fe(CN)_6_, and 2 mL of the 3% (*W*/*V*) PEI solution were prepared in 0.1 M HCl. Then, 10 mL of K_3_Fe(CN)_6_ and 2 mL of 3% PEI were mixed, 0.1488 g of KCl was dissolved in this mixture, and the resulting solution was heated up to 70 °C. Next, 10 mL of 10 mM of FeCl_3_.6H_2_O was injected to the latter at 1 mL/min for 10 min, maintaining the temperature at 70 °C. The resultant solution was vigorously stirred and heated up to refluxing temperature, after which it was refluxed for 30 min. After the sample temperature dropped to around 30 °C, the nanoparticles were separated by centrifugation (20,000 rpm, 15 min), Finally, the particles were washed with pure water twice and with ethanol once, and were stored in pure water for further investigation. Mass obtained = 31 mg.

#### 2.2.3. **T3**—PB/PEI Nanoparticles

A total of 10 mL of 5 mM of FeCl_3_.6H_2_O, 10 mL of 5.625 mM K_3_Fe(CN)_6_, and 2 mL of the 3% (*W*/*V*) PEI solution were prepared in 0.1 M HCl. These three solutions were mixed together and kept to form PB/PEI NPs for 60 h without stirring. Finally, the particles were separated by centrifugation (20,000 rpm, 15 min) and washed twice with water and once with ethanol. The purified particles were stored in pure water for further investigation. Mass obtained = 4 mg.

### 2.3. Synthesis of PB/PEI Nanoparticles by Custom Home-Made Vortex Process (**V1**)

The synthesis process was conducted in a custom-built vortex reactor vessel with an angle of 45°, as depicted in Scheme S1 (see Supplementary Information). The vessel tube was connected to a stepper motor that enabled the vessel to rotate at high speed under the influence of the power supply, thereby creating a vortex in the introduced solution and a lamellar flow within the vessel tube.

Firstly, 11.25 mM Na_4_Fe(CN)_6_.10H_2_O solution was prepared by dissolving the sodium ferrocyanide in 0.5% PEI (0.5 g/100 mL of 0.25 M HCl). Subsequently, 10 mL of this mixture and 10 mL of 10 mM FeCl_3_.6H_2_O were injected into the vessel tube, which was rotated at 1600 rpm for 1 h at room temperature while the solution was perfused at a rate of 10 mL/h using a peristaltic pump. Finally, the particles were supplemented with acetone (Carlo Erba, France, RE-Pure) (1:2 sample:acetone), separated by centrifugation (20,000 rpm, 15 min), and washed three times with water/acetone. The purified particles were stored in pure water for further investigation. Mass obtained = 15 mg.

### 2.4. Investigation of POD-like Catalytic Activity of the Nanoparticles

The POD-mimicking activity of the NPs (**Cb**, **C1**, **T1**, **T2**, **T3**, and **V1**) was examined using a (TMB)/H_2_O_2_ commercial solution as the substrate (Ref. T4444, Merck, Darmstadt Germany), which produces a soluble blue product by the action of HRP. In these assays, each type of NP was diluted serially with water in the wells of a flat-bottomed microplate (100 μL/well). Next, the wells were incubated with 100 μL of TMB enzyme substrate for 20 min at room temperature in the dark, 50 μL of 1 M H_2_SO_4_ was added to stop the reaction, and the absorbance was immediately measured at 450 nm using a FLUOstar Omega microplate reader (BGM Labtech, Ortenberg, Germany).

To investigate the steady-state kinetic behaviour of the **Cb**, **C1**, **V1**, **T1**, **T2**, and **T3** nanoparticles, each type of NP was processed as above, then 100 μL of substrate was added in each well, which included a fixed concentration of TMB and increasing concentrations of H_2_O_2_, and the absorbance was measured at 653 nm over time.

The reaction rates were plotted against the corresponding H_2_O_2_ concentrations using the Michaelis–Menten model (Equation (1)) and the Lineweaver–Burk reciprocal model (Equation (2)), which were employed to establish the maximum reaction velocity (Vmax) and Michaelis−Menten constant (Km).

(1) $$V_{0}=\frac{V_{max}\cdot \left[S\right]}{K_{m}}+\left[S\right]$$

(2) $$\frac{1}{V_{0}}=\frac{1}{V_{max}}+\frac{K_{m}}{V_{max}}\cdot \frac{1}{\left[S\right]}$$

To study the effect of sodium azide (NaN_3_), sulphuric acid (H_2_SO_4_), ethylenediaminetetraacetic acid (EDTA), and high concentrations of hydrogen peroxide (H_2_O_2_), four well-known inhibitors of the catalytic activity of HRP, on the PBNPs, catalytic activity assays were performed as above, but supplementing the TMB/H_2_O_2_ substrate solution with increasing concentrations of the corresponding inhibitor.

### 2.5. PB and PB/PEI Nanoparticle Characterisation

TEM images were recorded using a Jeol 1400 Plus Transmission Electron Microscope (JEOL, Akishima, Japan) operating at 100 kV accelerating voltage. Samples for the TEM measurements were deposited from suspensions on copper grids and allowed to dry before observation. Data were collected with a high-sensitivity sCMOS JEOL Matataki Flash camera (JEOL, Akishima, Japan). SEM-EDX were performed on an FEI Quanta FEG 200 instrument (Hillsboro, OR, USA). NP powders were deposited on an adhesive carbon film and analysed under high vacuum. The quantification of the heavy elements was carried out with the INCA software, with a dwell time of 3 µs.

FTIR spectra were recorded with a PerkinElmer Spectrum Two spectrophotometer (Waltham, MA, USA) with four acquisitions at a resolution of 4 cm^−1^. UV–Vis spectra were collected on a JASCO V-650 spectrometer (JASCO Corporation, Tokyo, Japan).

The crystal structural characteristics were investigated in the 2θ interval 10–70° at room temperature by XRD using a PANalytical X’Pert Powder analytical diffractometer mounted in a Debye–Scherrer configuration and equipped with Cu radiation (λ = 1.5418 Å) on powdered samples (Malvern Panalytical, Malvern, UK). The XRD patterns were analysed by pattern matching using the Fullprof Software (5.2) [24]. The crystallite size was calculated using the Williamson–Hall method, integrated into this software with a pseudo-Voigt Cox–Thompson function, accounting for instrumental broadening determined from a reference sample (Y_2_O_3_ powder annealed at 1400 °C for 36 h).

Electrochemical measurements were conducted utilising commercial screen-printed carbon electrodes (SPCE; DRP-110) and a multi-potentiostat (μStat 8000, Metrohm Dropsens, Oviedo, Spain) with DropView 8400 software. For this, 10 µL of the PBNPs or PB/PEI NPs (0.1 mg/mL in water) were applied to the surface of a cleaned SPCE working electrode and allowed to dry at 37 °C for 15 min. CV was then registered in the +1.2 to −0.2 V range using a scan rate of 50 mV/s, utilising 50 µL of 0.1 M KCl (pH = 4) as the electrolyte solution.

X-ray photoelectron spectrometry (XPS) experiments of the different nanomaterials were achieved with a Thermo K-alpha spectrometer equipped with a hemispherical analyser and micro focused (400 µm diameter microspot) monochromatic radiation (Al Kα, 1486.6 eV) operating under a residual pressure of 1 × 10^−9^ mbar. The pass energy was adjusted to 20 eV. To compensate for the charge effects occurring during the analysis, a dual beam charge neutralisation system (low energy electrons and Ar^+^ ions), which has the unique ability to provide consistent charge compensation, was used. The calibration of all spectra was based on the binding energy of a carbon 1 s orbital at 285.0 eV. The mathematical fitting was conducted with the Casa XPS software using a least squares algorithm and a nonlinear baseline (Shirley). The experimental curves peaks were fitted using a combination of Gaussian (70%) and Lorentzian (30%) distributions.

## 3. Results and Discussion

**Figure 1:**
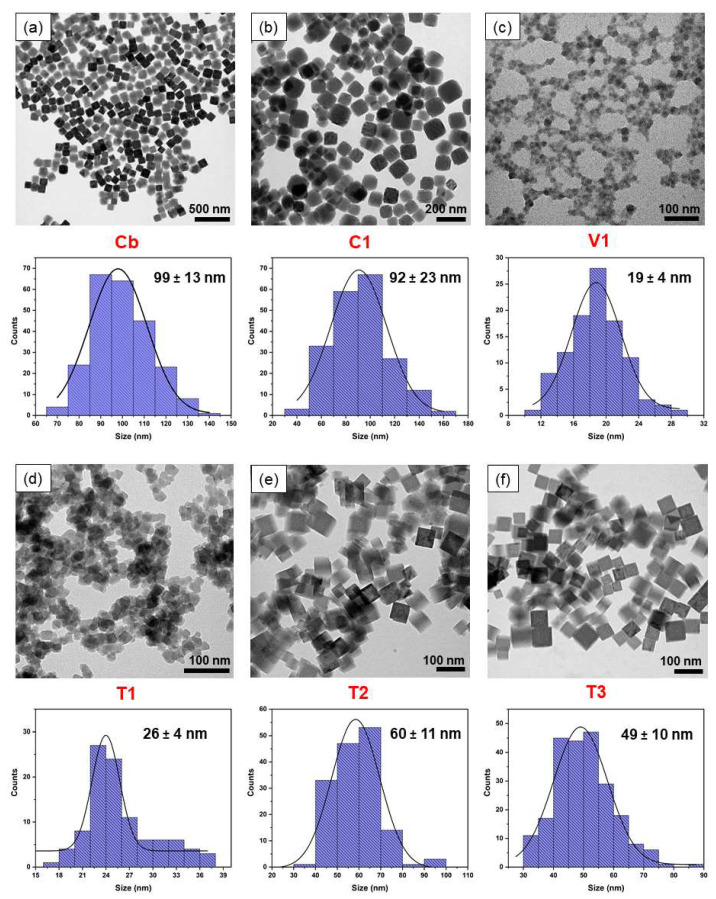
TEM images and size distribution of the (**a**) co-precipitately synthesised bare PBNPs Cb and (**b**) PB/PEI C1 nanoparticles; (**c**) V1 nanoparticles synthesised by the vortex technique; and the (**d**) T1, (**e**) T2, (**f**) T3 nanoparticles produced through a ferricyanide reduction technique.

A controlled coprecipitation technique was employed to synthesise the bare PBNPs, designated as **Cb**. In this technique, it is crucial to control the precursor concentration, injection rate, and the reaction medium temperature in order to obtain the desired particle size and shape. The manipulation of the aforementioned parameters, either collectively or individually, results in alterations to the shape, size, and Fe/Na. For instance, an increase in the precursor injection rates, while maintaining the other conditions constant, leads to a reduction in particle size and the generation of a broad range of particle sizes. Accordingly, these parameters were precisely maintained in accordance with the specifications outlined in Section 2.1. The **Cb** nanoparticles exhibited a cubic shape and a particle size of 99 ± 13 nm (edge/side length of the cubes), as illustrated in the TEM image (Figure 1a). The structural formula of **Cb** was determined to be Na_0.32_Fe[Fe(CN)_6_]_0.83_, calculated by the Fe/Na ratio in the EDX analysis (Table S1). A modification of this co-precipitation technique, which was used to synthesise **Cb**, was employed to synthesise in situ PB/PEI NPs (**C1**) by nucleating and growing the nanoparticles within an acidic (pH 1) PEI solution. The negatively charged Na_4_Fe(CN)_6_ is capable of attracting the positively charged PEI chains, facilitating the nucleation of PB/PEI NPs in the presence of Fe^3+^ ions. However, particle nucleation and growth were altered by the presence of the polymer in the particle synthesising medium. Consequently, the **C1** PB/PEI NPs exhibited a formula of Na_0.23_Fe[Fe(CN)_6_]_0.81_ and a cubic structure with an uneven edge surface and a broader range of particle sizes (92 ± 23 nm, as shown in Figure 1b). A custom-made vortex reactor (Scheme S1) was employed to synthesise the **V1** PB/PEI NPs. To the best of our knowledge, this is the first instance of this protocol being used to synthesise Prussian blue-type nanoparticles. Nevertheless, this technique has already been applied to a number of distinct nanoparticle types with prominent examples including co-precipitated poly(vinyl pyrrolidine)-coated superparamagnetic magnetite nanoparticles [25]. In this process of **V1** synthesis, droplets of the precursors, PEI/Na_4_Fe(CN)_6_ and FeCl_3_, were subjected to vortexing inside the reactor, which was rotated at 1600 rpm. The shear stress applied to the precursor solution led to the formation of smaller ill-defined nanoparticles (19 ± 4 nm; Figure 1c). Furthermore, the EDX analysis revealed that the nanoparticles exhibited the lowest alkali metal percentage, with a formula of Na_0.11_Fe[Fe(CN)_6_]_0.78_.

Three types of particles with different shapes and sizes were synthesised by the thermal reduction of [Fe(CN)_6_]^3−^ in the presence of PEI, which is attracted electrostatically to [Fe(CN)_6_]^3−^. The [Fe(CN)_6_]^3−^ is then reduced to [Fe(CN)_6_]^4−^, followed by the formation of PB/PEI NPs after complexation with Fe^3+^. PB/PEI NPs **T1** with formula K_0.15_Fe[Fe(CN)_6_]_0.78_ were synthesised with high precursor concentrations (see Section 2.2. for details), resulting in the generation of highly aggregated non-uniform cubic/spherical particles with sizes of 26 ± 4 nm (Figure 1d). By using lower precursor concentrations and increasing the ionic strength by adding KCl to the PB/PEI NPs, we obtained **T2** particles, K_0.32_Fe[Fe(CN)_6_]_0.83_, which were cubic-shaped and 60 ± 11 nm in size (Figure 1e). The room temperature reduction of [Fe(CN)_6_]^3−^ into [Fe(CN)_6_]^4−^ and reaction without refluxing over 60 h produced PB/PEI NPs **T3** (Figure 1f), which displayed a cubic shape, 49 ± 10 nm size, and the formula of K_0.14_Fe[Fe(CN)_6_]_0.79_. In the case of **T3**, a slower kinetic rate synthesis process was trialled to ascertain the viability of synthesising PB/PEI without refluxing. However, under the aforementioned conditions in Section 2.2, it took nearly 60 h to generate a PB crystalline phase, which is considerably longer than the refluxing approach, which only takes 30 min. It is worth noting that the synthesis of the nanoparticles by this thermal reduction protocol is highly sensitive to the precursor saturation/volume, pH, and polymer percentage. Thus, for these three types of nanoparticles, particularly for **T1**, the solute concentration may exceed the critical nucleation point when subjected to high solute concentrations. This can result in the rapid formation of a high percentage of small nuclei through explosive nucleation. In general, with regard to the considered NP types, the particles synthesised with low kinetic rates for a long-time reaction process tended to result in higher particle sizes. However, under conditions of continuous precautionary feeding, the NPs could be grown in a controlled manner compared to short-time, faster kinetic rate synthesis methods.

Of the three types of particle synthesis protocols, the vortex protocol shows considerable promise for scaling up through the use of continuous precursor injection and continuous product withdrawal from the vortex reactor. The co-precipitation technique presents a significant challenge when attempting to scale up, as the parameters must be optimised for different batch sizes. However, the sensitivity of the particle size and shape, coupled with the necessity for an external energy input to drive the temperature evolution, makes thermally-synthesised particles particularly unfavourable for large-scale production.

**Figure 2:**
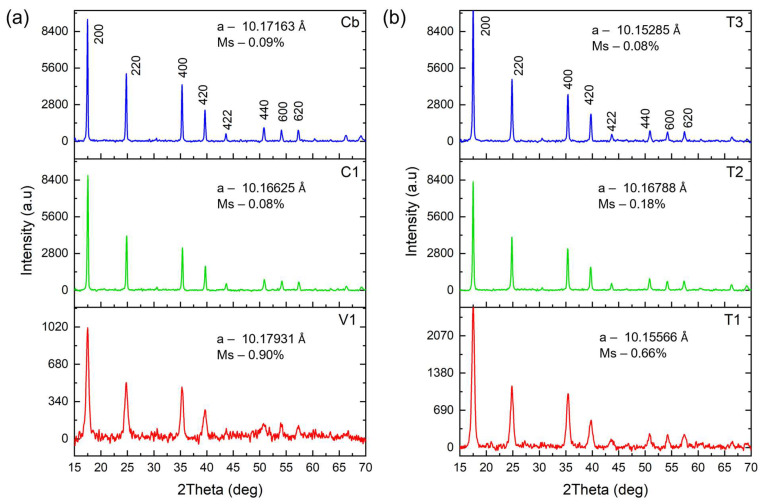
XRD patterns of the (**a**) bare PBNPs—**Cb**, in situ coprecipitated PEI/PB NPs—**C1**, and **V1** synthesised by vortex; (**b**) **T1**, **T2**, and **T3** synthesised by the thermal process. a—lattice parameter; Ms—microstrain.

The XRD matching pattern for all six types of particles, as seen in Figure 2a,b, can be attributed to the standard PB crystal JCPDS card 73-0689 with a cubic crystal system with an Fm-3 m space group, and the main peaks around 2θ of 17.452°, 24.778°, 35.326°, 39.660°, 43.630°, 50.820°, 57.337°, 66.322°, and 69.172° [26,27]. The crystallinity of the PEI-mediated in situ precipitated **C1** nanoparticles remained roughly unchanged compared to the bare **Cb** NPs (Figure 2a). The diffraction patterns were refined using the pattern matching method to obtain accurate information of the crystalline materials (see Table S2). The lattice parameter-a of the **C1** NPs (10.16625 Å) was smaller than that of the bare PBNPs (10.17163 Å). This reduction can be attributed to the external pressure exerted by the PEI polymer during the nucleation and growth of the NPs, which compresses the crystal lattice [28,29]. The electrostatic interaction and Fe-N coordination between the crystal and PEI can lead to a compressive contraction of the lattice planes, further contributing to the overall lattice compression. It is noteworthy that the mean microstrain of these two particles exhibited nearly identical values of 0.09% and 0.08%, indicating a minimal prevalence of crystal defects in their structure. This observation lends support to the view that the in situ synthesis of PBNPs does not result in any substantial crystal defects or microstrains. On the other hand, the **V1** NPs exhibited the highest lattice parameter (10,17931 Å), together with the highest microstrain of 0.90% among all particles. The reduced particle size of **V1** resulted in the constrained arrangement of a high number of atoms at the surface, leading to elevated surface energies. This effect contributed to an increase in crystal defects and a broad distribution of microstrains [30]. Moreover, the rapid nucleation of nanoparticles in this vortex protocol gives rise to enhanced crystal distortion, characterised by elevated microstrains, and a notable reduction in crystallinity (Figure 2a).

The XRD patterns of the particles that were synthesised by thermal reduction are shown in Figure 2b. The microstrain value and the lattice parameter of these nanoparticles were 0.66%/10.15566 Å, 0.18%/10.16788 Å, and 0.08%/10.15285 Å for **T1**, **T2**, and **T3**, respectively. The same phenomenon of high microstrains in smaller particle sizes was demonstrated for the **T1** nanoparticles, which exhibited 0.66% of microstrain. Furthermore, the combination of temperature with high reaction kinetic rates was a contributing factor to the high microstrain observed in both **T1** and **T2** compared to the microstrain values of the **T3** nanoparticles (0.08%), which were synthesised at room temperature with slower kinetic rates. The crystallite sizes of the particles were estimated by pattern matching to be 91, 62, 23, 18, 73, and 45 nm for **Cb**, **C1**, **V1**, **T1**, **T2**, and **T3**, respectively (see Table S2).

**Figure 3:**
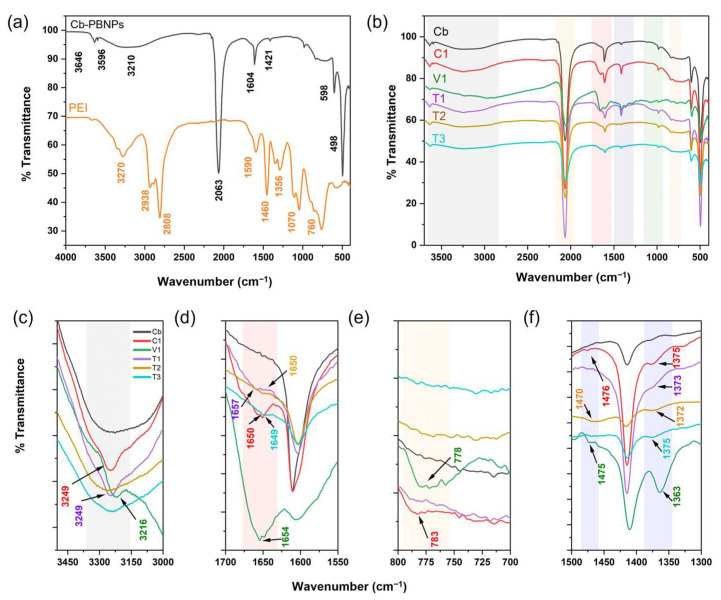
FTIR spectra of the (**a**) bare PBNPs **Cb** and PEI, (**b**) **Cb**, and **C1**, **V1 T1**, **T2**, and **T3** PB/PEI NPs. (**c**–**f**) Magnification of the considerable peaks in the spectrum.

As illustrated in Figure 3a, the conventional FTIR vibrational peaks of the bare PBNPs **Cb** were attributed to the bands at 3210/3646, 3596, 1604, and 1421 cm^−1^ for the OH stretching vibrations in the absorbed H_2_O (bonded OH/free OH), OH stretching vibrations in the crystallised H_2_O, the OH bending vibrations in the absorbed H_2_O, and OH bending vibrations in the crystallised H_2_O, respectively [31]. An intense band for the CN stretching vibration could be observed at 2063 cm^−1^, accompanied by the Fe^2+^-CN stretching and the bending vibrational bands at 598 and 498 cm^−1^, respectively [10,32]. All of the aforementioned vibrational bands were present in all of the PB/PEI NPs (Figure 3b and Table S3), providing evidence for the synthesis of PB. In addition, a number of peaks were characteristic of only the PB/PEI NPs. A vibrational band appeared at 3249 cm^−1^ in both **C1** and **T1** and at 3216 cm^−1^ in **V1** (Figure 3c). This peak can be attributed to the N-H stretching vibrational band that appeared due to the presence of PEI. However, this band might overlap with that of the OH stretching band, which made its identification challenging in **T2** and **T3**. A weak band around 1650 cm^−1^ in all of the PB/PEI particles could be assigned to the bending vibrations of N-H bonding (Figure 3d) [32,33]. This band was red-shifted compared to the pure PEI polymer and could be clearly identified in the **C1** and **V1** particles at 1650 cm^−1^ and 1654 cm^−1^, respectively. Furthermore, **C1** and **V1** exhibited the N-H wagging-bending vibrational peak at 783 and 778 cm^−1^, respectively (Figure 3e). The C-H bending vibrational band was also observed at approximately 1375 cm^−1^ for all of the PB/PEI NPs, and another C-H bending vibrational band could be seen in **Cb**, **V1**, and **T2** at 1476, 1475, and 1470 cm^−1^, respectively (Figure 3f) [34,35]. The aforementioned data indicate that the presence of PEI in the particles was more pronounced for the in situ coprecipitated and vortex particles than in the particles produced through the thermal process.

**Figure 4:**
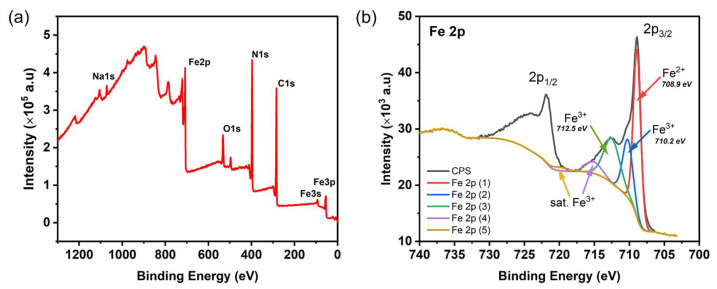
(**a**) XPS elemental survey spectra obtained for the PBNPs (**Cb**). (**b**) High resolution XPS spectra acquired for Fe 2p in **Cb**.

XPS characterisation of the bare PBNPs **Cb** revealed the typical binding energies (Figure 4a). The most prominent XPS peaks at 55.1 eV, 93.1 eV, 285.0 eV, 398.1 eV, 532.4 eV, and 708.9 eV, and 1073.2 eV were associated with the binding energies of the electrons of Fe 3p, Fe 3 s, C 1 s, N 1 s, O 1 s, Fe 2p, and Na 1 s, respectively [36]. Similar XPS peaks were obtained for **C1** and **V1**. However, for the **T1**, **T2**, **T3** particles, a K 2p peak around 294.2 eV was observed instead of the Na 1 s peak at 1073.2 eV (Figure S1). High-resolution XPS analysis was conducted on the Fe 2p electrons in the particles in order to obtain the valence information of Fe, which is the main component involved in the POD-like activity in PB. The XPS spectra of **Cb** particles were deconvoluted into five distinguishable peaks, as illustrated in Figure 4b. The three principal peaks, situated at 708.9 eV, were indicative of the Fe^2+^ 2p_3/2_ valence state, while the peaks at 710.2 eV and 712.5 eV were indicative of the Fe^3+^ 2p_3/2_ valence states, respectively. Two additional satellite peaks were identified at 715.1 eV and 720.0 eV [37,38]. The XPS spectra of the five types of PB/PEI NPs exhibited a similar pattern, with five deconvolution peaks situated at approximately the aforementioned positions. The valence information obtained from XPS revealed that the Fe^2+^/Fe^3+^ ratio was 0.94, 0.88, 1.37, 1.31, 1.28, and 1.25, calculated according to the above-mentioned three peaks in **Cb**, **C1**, **V1**, **T1**, **T2**, and **T3**, respectively. The synthesis protocols, which included controlled co-precipitation, thermal reduction, and the vortex method, demonstrated a low to high Fe^2+^/Fe^3+^ ratio trend.

**Figure 5:**
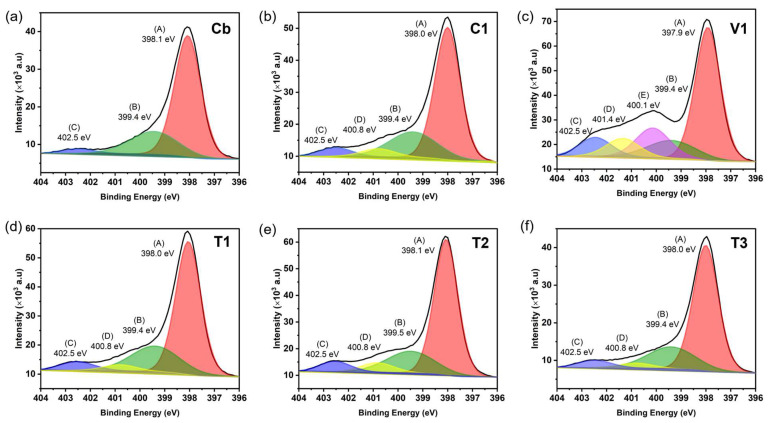
High resolution XPS spectra for N 1 s of the (**a**) **Cb**, (**b**) **C1**, (**c**) **V1**, (**d**) **T1**, (**e**) **T2**, and (**f**) **T3** nanoparticles.

High resolution narrow scan XPS analysis was performed on the N 1 s electrons in the particles to determine the presence of PEI on their surface. The two main deconvolution peaks at 398.1 and 399.4 eV in the **Cb** particles, as shown in Figure 5a, corresponded to the C≡N bond coordinated to Fe^2+^ and Fe^3+^ in the PB structure, respectively [18,39,40]. These two peaks also appeared in all types of PB/PEI NPs, but shifted ±1 eV (Figure 5b–f). The minor deconvolution peak in **Cb** at 402.5 eV may be due to the oxidisation of the N atom [40]. Nevertheless, in comparison to the three deconvolution peaks (A, B, C) observed in the N 1 s XPS spectra of **Cb**, four deconvolution peaks (including an additional D) were present in **C1**, **T1**, **T2**, and **T3**, while five deconvolution peaks (additional D and E) appeared in **V1**, attributed to the incorporation of PEI. The peak D at 400.8 eV in **C1**, **T1**, **T2**, and **T3** and at 401.4 eV in **V1** can be attributed to the NH from the PEI. Additionally, the peak at 400.1 eV in **V1** can be attributed to the amine N-R/N-R2 groups in the PEI [23,41,42,43]. Furthermore, the protonated amine peaks were also anticipated to be present at approximately 402.1 eV, which may overlap with the peak at 402.5 eV [18]. Nevertheless, the increase in the peak area at 402.5 eV indicates the additional N (from PEI) in all PB/PEI NPs, confirming the presence of PEI. It can be reasonably inferred that the increase in N concentration attributable to PEI was approximately 5.3, 6.5, 4.0, 3.7, and 25.3% in **C1**, **T1**, **T2**, **T3**, and **V1**, respectively, compared to **Cb**.

The electrochemical behaviour of the PBNPs and PB/PEI NPs was studied by cyclic voltammetry by drying 10 µL of the nanoparticles (0.1 mg/mL in water) on the working electrode of an SPCE and using 0.1 M KCl (pH 4) as the electrolyte. The conventional electrochemical switching of oxidation (partially/fully) and reduction of PB into Berlin green (BG)/Prussian yellow (PY) and Prussian white (PW), respectively, can be explained by the following chemical equations [14,15,44]. In these equations, A represents Na or K, which originate from the synthesis, and B represents K, which originates from the electrolyte.

(PB)     AFe^3+^[Fe^2+^(CN)_6_] → Fe^3+^[Fe^3+^(CN)_6_] + A^+^ + e^−^     (PY) Oxidation (1)

(PB)     AFe^3+^[Fe^2+^(CN)_6_] + B^+^ + e^−^ → ABFe^2+^[Fe^2+^(CN)_6_]     (PW) Reduction (2)

**Figure 6:**
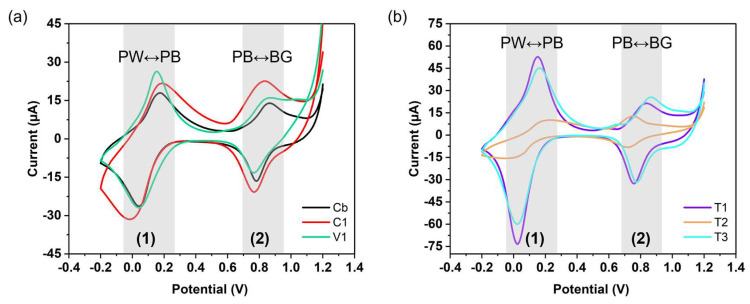
Cyclic voltammetry for (**a**) **Cb**, **C1**, **V1** and (**b**) **T1**, **T2**, **T3**. Scan rate—0.1 V s^−1^, electrolyte 0.1 M KCl at pH 4.

The conventional electrochemical behaviour of PB was clearly evident in all nanoparticles, which showed two pairs of quasi-reversible peaks at potentials between 0 and 0.23 V for PB to PW (redox couple 1) and 0.71 V and 0.86 V for PB to BG (redox couple 2; BG—{Fe(III)_3_[Fe(III)(CN)_6_]_2_[Fe(II)(CN)_6_]}^−^; Figure 6) [15]. When comparing **Cb** and **C1**, which were synthesised by co-precipitation, **C1** displayed larger ΔE and higher peak currents for the first redox couple, indicating lower reversibility but enhanced electron transfer (Table S4). This was attributed to the protonated amine groups in the polymer, which acted as a bridge to transfer electrons and supported the increase in current, but at the same time affected the PB structure. Moreover, the elevated symmetry of the peak shapes representing the first redox couple in Cb suggests that the electron transfer processes in Cb are more reversible than in C1. The **V1** particles were also nucleated by precipitation, but through the vortex mixing protocol. The resulting particles were smaller than **Cb** and **C1** and displayed smaller ΔE and higher peaks than **Cb** for the first redox pair. In contrast, the redox potentials observed in the second redox couple were nearly identical for **Cb**, **C1**, and **V1** (Figure 6a), reaching the perfect reversibility region below 59 mV (for a one-electron transfer), but the peaks were wider and lower for **V1**. This was probably due to the high polymer loading in **V1** (see XPS data in Figure 5c).

Figure 6b depicts the CVs of PB/PEI NPs produced by the thermal reductive protocol, which displayed two redox waves similar to those observed for the co-precipitated bare particles **Cb**. It can be generally expected that, for a fixed mass of NPs, smaller particles will exhibit a larger total surface area, which will in turn contribute to more efficient electron transfer. According to the XRD data (see Table S2), the crystal size of the NPs decreased in the order of **T2** > **T3** > **T1**, and the recorded current also increased in the manner of **T2** < **T3** < **T1**. On the other hand, the ΔE of the first redox couple indicated good reversibility for **T1** and **T3** compared to **T2**. However, the second redox couple was more reversible in **T2**, which also demonstrated the symmetry of the peak shapes.

In all cases, but especially in the case of **T1** and **T3**, the peaks of the first redox wave were higher than those in the second wave. In **Cb**, **C1**, **V1** and **T1**, **T2**, **T3**, the alkali atoms Na and K, respectively, were intercalated into the vacancies of the PB crystal lattice. Additional K^+^ from the electrolyte can intercalate into the lattice when reducing PB to PW, while the alkali atoms can be removed from the crystal lattice when oxidising PB to BG. Accordingly, in all particles, the first redox couple may be more efficiently accessible to the electrolyte, leading to faster diffusion and higher current, as shown in Figure 6a,b.

**Figure 7:**
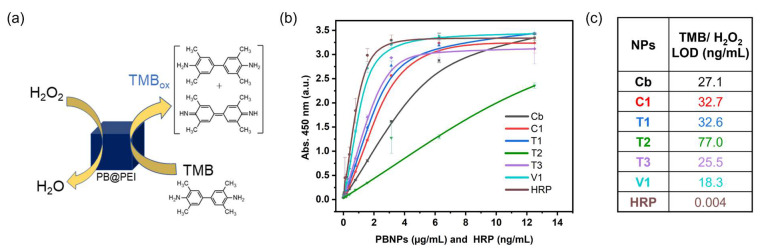
(**a**) Schematic diagram to illustrate the POD-like activity of the PB/PEI NPs. (**b**) Activity assay for increasing concentrations of each type of nanoparticle and HRP, incubated with TMB/H_2_O_2_ for 20 min and stopped with acid before measuring the absorbance at 450 nm. (**c**) LOD values for each nanoparticle type in (**b**).

The POD-mimicking catalytic activity of the PBNPs and PB/PEI NPs was investigated by incubating them with a chromogenic ready-to-use (TMB)/H_2_O_2_ substrate solution. All nanoparticles exhibited POD-mimicking catalytic activity, reducing H_2_O_2_ to H_2_O and oxidising the colourless TMB_Red_ to its blue redox intermediate TMB_Red_/TMB_Ox_, as HRP does (Figure 7a) [45]. The reaction was then stopped by adding sulphuric acid, which produced yellow fully oxidised TMB_Ox_, which was measured at 450 nm. Figure 7b presents the signals measured as a function of nanoparticle type and concentration up to 12.5 µg mL^−1^ (signal saturation was reached at higher concentrations for all nanoparticles). Figure 7c depicts the limit of detection (LOD) for each curve, which indicates the lowest concentration of nanoparticle that could be detected. At first glance, it was evident that all PB/PEI NPs except **T2** produced higher signals than **Cb**, with higher catalytic activity correlating with lower LOD. However, **Cb** displayed a lower LOD than most PB/PEI NPs (27 ng/mL). This was attributed to PEI, which could alter or shield the PB catalytic sites, making it harder to detect low particle concentrations but providing protonated amine groups that could also facilitate electron transfer, especially at high particle concentrations.

When the two types of nanoparticles produced by coprecipitation were compared, **C1** and **Cb** had an approximately similar size, however, **C1** exhibited a higher catalytic activity but slightly higher LOD. **C1** showed a lower reversibility of the first redox wave in the CV and lower colloidal stability, thus a lower exposure of active surface to the medium (zeta potential 0.07 ± 2.5 mV and −46.8 ± 6.5 mV for **C1** and **Cb**; Figure S2). The EDX results indicated that **C1** displayed slightly higher Fe to alkali content (88.63% and 85.06% Fe for **C1** and **Cb**, respectively; see Table S1), which could result in more catalytic sites, while the Fe^2+^/Fe^3+^ ratio was lower (0.88 versus 0.94). The main difference seemed to be the PEI presence in **C1**, with an estimated 5.3% of nitrogen derived from the PEI, as determined by XPS.

On the other hand, **V1** displayed the highest catalytic activity and lowest LOD observed. It was anticipated that smaller particle sizes should result in enhanced catalytic activity due to the increased surface area [46]. **V1** was the smallest nanoparticle tested (19 ± 4 nm apparent size and 23 nm crystalline size; Table S2) and was also characterised by high colloidal stability (zeta potential 38.0 ± 4.4 mV) and the highest microstrain (0.9%), Fe to alkali content (94% Fe), Fe^2+^/Fe^3+^ ratio (1.37), and PEI incorporation (25.3%). Thus, the reason for the high catalytic activity of **V1** was presumably the combination of small particle size, the increased amount of well exposed active surface, elevated number of highly active catalytic sites, and efficient PEI-driven wiring.

For the particles produced via thermal reduction, **T3** demonstrated higher catalytic activity than **T1**, and **T2** exhibited the lowest catalytic activity among all particles. Again, smaller particles worked better. The bad performance of **T2** was correlated to the low peak current and reversibility observed by CV. Of these three types of particles, **T2** also displayed the lowest Fe content, but the numbers were still slightly larger than those of **Cb**, which performed better. Furthermore, the concentration of PEI in **T2** was between those in **T1** and **T3**.

Globally, the results suggest that none of the parameters studied was solely responsible for nanoparticle catalytic activity. However, a combination of small size, high iron content, and high Fe^2+^/Fe^3+^ ratio seemed determinant for particles displaying high catalytic activity. The incorporation of the aminated polymer could facilitate electron transfer between the nanocatalyst and the TMB/H_2_O_2_ system in some cases, but also disrupt or shield the PB catalytic active sites in others. Finally, while, in general, bad catalysts produced distorted CVs and good catalysts produced larger and more reversible peaks, peak height and reversibility were not useful to solely define catalyst efficiency. Independently of this, the LODs calculated for all of the nanoparticles were higher than those obtained for HRP, which was a better catalyst.

**Figure 8:**
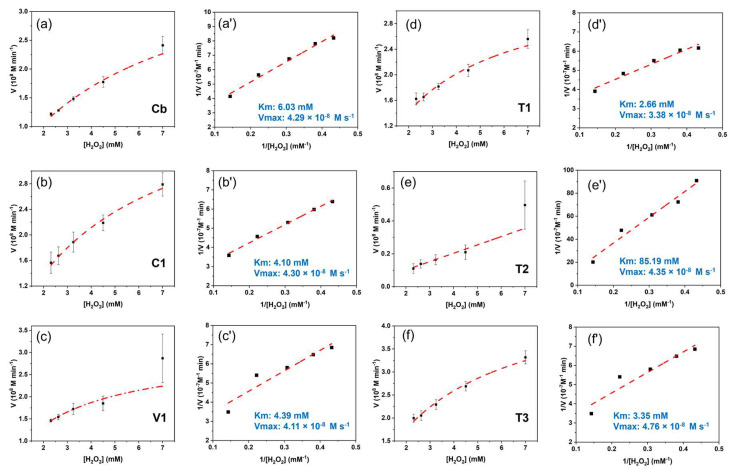
Kinetics of the catalysis of the particles. (**a**–**f**) The Michaelis–Menten model and (**a’**–**f’**) Lineweaver–Burk reciprocal model for the POD-like catalytic activity of the **Cb**, **C1**, **V1**, **T1**, **T2**, and **T3** nanoparticles, respectively.

The steady-state kinetic behaviour of the nanoparticles was investigated further, this time using a fixed concentration of TMB and increasing concentrations of H_2_O_2_ (Figure 8). The Michaelis constant (Km) and maximal velocity (Vmax) were calculated by utilising the Michaelis–Menten model (Figure 8a–f) and the Lineweaver–Burk reciprocal model (Figure 8a’–f’), respectively. The Km values were found to be 2.66, 3.35, 4.10, 4.39, and 6.03 mM for **T1**, **T3**, **C1**, **V1**, and **Cb**, respectively, while **T2** exhibited a higher value of 85.19 mM (see Table S5). Since Km defines the affinity between H_2_O_2_ and the catalyst [47], the values obtained revealed a higher H_2_O_2_ affinity towards PB/PEI NPs than to **Cb**, with the exception of **T2**. Although the highest affinity was obtained in the low particle size range (<50 nm), the smallest **V1** displayed an unexpected low affinity for H_2_O_2_. On the other hand, the Vmax was approximately of the same order for all types of nanoparticles (4.76 × 10^−5^ mM s^−1^, 4.35 × 10^−5^ mM s^−1^, 4.30 × 10^−5^ mM s^−1^, 4.29 × 10^−5^ mM s^−1^, 4.11 × 10^−5^ mM s^−1^, 3.38 × 10^−5^ mM s^−1^ for **T3**, **T2**, **C1**, **Cb**, **V1**, and **T1**, respectively). While Km and Vmax are frequently employed to evaluate the catalytic activity of PBNPs, the LOD value provides a simpler and more direct measure of their catalytic performance (Table S5). The **V1**, **T1**, and **T3** nanoparticles demonstrated the optimal POD-like catalytic performance across the evaluated parameters, along with the lowest LOD, the lowest Km, and the highest Vmax, respectively.

The catalytic activity of the PBNPs can be predominantly attributed to the redox cycling of the oxidative and reductive states of PB, coupled with electron transfer between the PB and TMB/H_2_O_2_. As proposed by Feng et al., in our system, PB could undergo a valence band pathway by transferring an electron to reduce H_2_O_2_ (causing PB oxidation), then accepting an electron from TMB (oxidising TMB and reducing oxidised PB). An alternative hypothesis is that the process of the conduction band pathway occurs when an electron is transferred from TMB to PB or pre-oxidised PB (causing the reduction of PB or pre-oxidised PB). This is followed by the release of an electron, which reduces H_2_O_2_, while oxidising PB or reduced state PB. Furthermore, as hypothesised by Komkova et al., PB initially reacts with TMB by reducing to PW and oxidising TMB, then reduces the H_2_O_2_ by oxidising PW [48,49].

Conversely, the iron valence states of PB have the potential to generate reactive oxygen species (ROS), which can influence the catalytic reaction, particularly in particle modifications and/or with external energy. However, it should be noted that PB is also capable of scavenging ROS [15,48,50]. Nevertheless, understanding the precise mechanism is challenging because of the complexity and incomplete elucidation of its various operational processes. Moreover, it is noteworthy to mention that the chromogenic agent TMB suffers from a lack of specificity to be applied in deep studies of the POD mechanisms [51].

**Figure 9:**
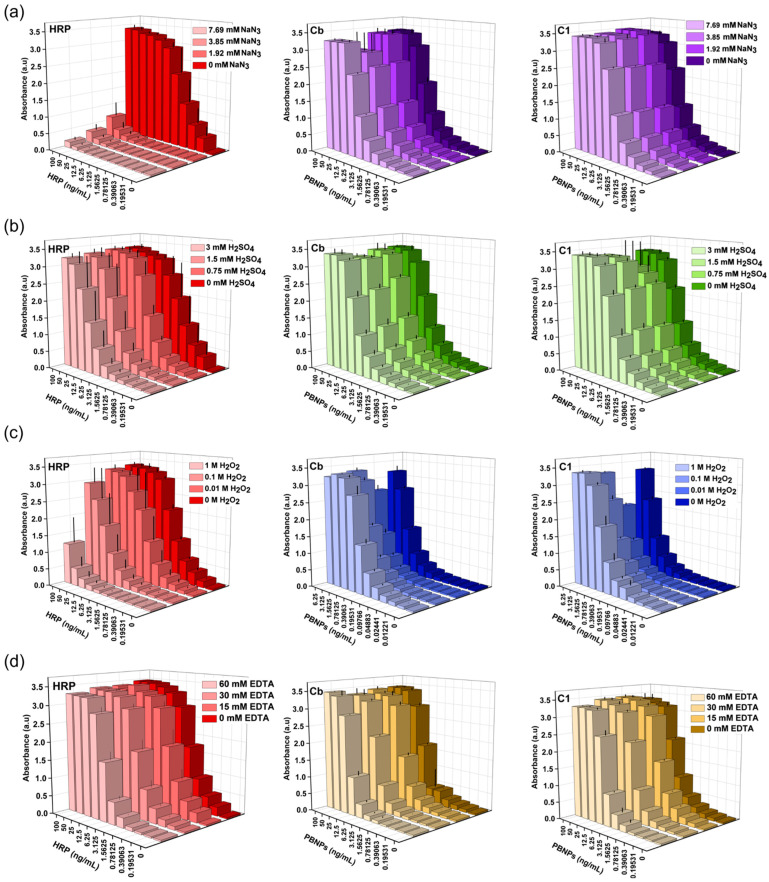
Effect of different inhibitors in the catalytic POD-like activity of **HRP**, **Cb**, and **C1**. Activity assay carried out for increasing catalyst concentrations in the substrate solution supplemented with (**a**) 7.69, 3.85, 1.92, and 0 mM of NaN_3_; (**b**) 3, 1.5, 0.75, and 0 M of H_2_SO_4_; (**c**) 1, 0.1, 0.01, and 0 mM of H_2_O_2_; (**d**) 60, 30, 15, and 0 mM of EDTA.

It is well-known that HRP activity is impaired in the presence of a number of inhibitors [52,53,54]. The POD-like activity of all synthesised particles was evaluated in the presence of a range of HRP inhibitors including NaN_3_, H_2_SO_4_, H_2_O_2_, and EDTA. Figure 9 depicts the evolution of the blue colour resulting from the oxidation of TMB, which was catalysed by the HRP or nanozymatic PBNPs (**Cb** and **C1**) at varying concentrations in the presence of the aforementioned inhibitors at different concentrations.

Both **Cb** and **C1** exhibited highly stable catalytic behaviour in the presence of 1.92–6.79 M NaN_3_ in comparison to the extreme inhibition caused to HRP (Figure 9a). The same was observed for **T1**, **T2**, **T3**, and **V1** (Figure S3), without any impact of the POD-like activity. This indicates that the coordination complex of PB, or its redox Fe^2+^/Fe^3+^system, is not influenced by NaN_3_. The catalytic activity and kinetics of HRP are also highly sensitive to acidic pH, which alters the enzyme structure and deactivates its active sites (Figure 9b-HRP) [18]. However, POD-like deactivation was not observed for the PBNPs and PB/PEI NPs (Figure 9b and Figure S4). On the contrary, when stored in basic solutions these particles tend to degrade, and the rate of degradation depends on the strength of the basicity and the storage time.

Figure 9c illustrates the gradual reduction in the catalytic activity of HRP observed when the TMB/H_2_O_2_ substrate solution was supplemented with increasing H_2_O_2_ concentrations of up to 1 M. This can be attributed to the formation of inactive enzymatic species, E*_x_*, leading to a reduction in catalytically active enzymatic sites [52]. It is noteworthy that for **Cb** and **C1**, as illustrated in Figure 9c, the catalytic activity was not only retained, but increased proportionally to the H_2_O_2_ concentration (see also Figure S5). This implies that the commercial TMB/H_2_O_2_ system, which is produced for HRP detection, includes a concentration of H_2_O_2_ far from optimal for PB maximal efficiency.

In contrast, the **Cb** bare PBNPs demonstrated higher catalytic inhibition by EDTA than HRP (Figure 9d). This was particularly evident at particle concentrations below 25 ng/mL in high EDTA levels. EDTA is a potent metal chelating that binds to Fe^2+^ and Fe^3+^ centres through its amine and carboxylic groups, forming complexes [55]. This results in the reduction of redox-active catalytic metal sites and POD-like activity in the PBNPs. Nevertheless, the PEI-modified particles **C1**, **T1**, and **V1** (see Figure S6) exhibited a lower impact on their activities. This could be due to the presence of the aminated PEI, which may repel EDTA to a certain extent, reducing the chelation of the metal centres.

Globally, these results show that PBNPs and PB/PEI NPs are more resistant than HRP to at least a number of potential inhibitors.

## 4. Conclusions

A range of PB/PEI NPs were synthesised using both novel/modified (**C1**, **T3**, **V1**) and conventional (**Cb**, **T1**, **T2**) synthesis protocols, yielding particles with sizes ranging from approximately 19 to 92 nm and diverse shape combinations. The successful formation of a cyano-bridged coordination network, adopting a cubic crystal structure with an Fm-3 m space group, was confirmed in all nanoparticles, consistent with the PB framework, as supported by the FTIR and XRD analyses. The **V1** and **T1** PB/PEI NPs, synthesised through high kinetic rate protocols and possessing smaller sizes, exhibited greater microstrains compared to the other NPs. The presence of PEI on the nanoparticle surfaces was confirmed using high-resolution narrow scan XPS of the N 1 s electrons, along with FTIR spectroscopy. CV analysis clearly revealed conventional redox behaviour in all types of synthesised PBNPs. However, the PB:BG redox couple exhibited higher reversibility compared to the PB:PW redox couple in all nanoparticles, except for those synthesised using the **T3** protocol, where the highest reversibility was observed in the PB:PW redox couple.

The POD-like activity of the particles was characterised using the TMB/H_2_O_2_ colorimetric system by determining the LOD across a range of particle concentrations. All particles exhibited catalytic activity consistent with POD-like properties, with the **V1** particles displaying the highest activity and the lowest LOD (18.3 ng/mL). This enhanced activity is likely attributable to the **V1** particles’ smaller size, higher microstrains, high colloidal stability, and higher Fe% compositional features, which collectively increased the exposure of active sites and improved the catalytic efficiency. In general, the kinetic studies demonstrated that the PEI-mediated synthesised PB nanoparticles exhibited a significantly higher affinity for H_2_O_2_ than the bare PBNPs, highlighting their potential for enhanced catalytic applications. Although the nanoparticle activity could not be predicted by studying a single parameter, higher activity was observed for NPs of smaller size, higher Fe content, and higher Fe^2+^/Fe^3+^ratio. In this context, PEI incorporation seemed to affect the PB structure and/or activity to a certain extent, which worsened the LOD of the PB/PEI NPs compared to the PBNPs, but provided electron wiring, granting larger signals at medium-to-high NP concentrations. Interestingly, while Km and Vmax are often used to characterise PBNPs, they were less useful in directly classifying the PB/PEI NPs, for which the LOD in the activity assay was more informative.

Furthermore, catalytic inhibition studies revealed that all PB/PEI NPs demonstrated excellent stability against common catalytic inhibitors of HRP such as NaN_3_, H_2_SO_4_, and concentrated H_2_O_2_. However, the PBNPs in **Cb** exhibited an inhibition of their POD-like activity in the presence of EDTA, due to the chelation of Fe^2^⁺ and Fe^3^⁺ centres. Notably, some of the PEI-modified particles (**C1**, **T1**, and **V1**) showed reduced inhibition by EDTA compared to **Cb**, indicating a lower degree of metal centre chelation, thereby enhancing their resistance to inhibition. The synthesised PB/PEI NPs demonstrated significant POD-like activity and enhanced chemical stability compared to HRP, positioning them as promising candidates for nanozymatic applications. This study contributes valuable insights into the properties and performance of PB/PEI NPs across a defined range of particle sizes, facilitating the informed selection of suitable PB/PEI NPs for future biosensor applications.

## Data Availability

The original contributions presented in this study are included in the article/Supplementary Material. Further inquiries can be directed to the corresponding author(s).

## Supplementary Materials

The following supporting information can be downloaded at: https://www.mdpi.com/article/10.3390/nano15010041/s1. Scheme S1: Schematic illustration of the vortex reactor, Table S1: Metal composition and the synthesis information of synthesised PBNPs and PB/PEI NPs, Table S2: Crystallite size, microstrain, and lattice parameter of the PBNPs and PB/PEI NPs, Table S3: FTIR peaks analysis, Table S4: Cyclic voltammetry peak analysis, Table S5: Catalytic activity of the particles—LOD and kinetic parameters, Figure S1: XPS elemental survey spectra for PBNPs and PB/PEI NPs, Figure S2: Zeta potential analysis, Figures S3–S6: Inhibition in the catalytic activities of the synthesised nanozymatic PB/PEI NPs (**T1**, **T2**, **T3** and **V1**) in the presence of NaN_3_, H_2_SO_4_, high concentrations of H_2_O_2_ and EDTA, respectively.
