# Supplementary material for: Peroxidase (POD) Mimicking Activity of Different Types of Poly(ethyleneimine)-Mediated Prussian Blue Nanoparticles
Source: Nanomaterials (Basel). 2024 Dec 29;15(1):41. doi: 10.3390/nano15010041 (PMC11722672; doi:10.3390/nano15010041)
Supplement: Supplementary file 1 [file nanomaterials-15-00041-s001.zip › nanomaterials-3369484-supplementary.pdf]

Supplementary Information

Peroxidase (POD) Mimicking Activity of Different Types of Poly(ethyleneimine)-Mediated Prussian Blue Nanoparticles

Udara Bimendra Gunatilake,[a*] Briza Pérez-López,[b] Maria Urpi,[b] Judit Prat-Trunas,[b,c] Gerard Carrera-Cardona,[b] Gautier Félix,[a] Saad Sene,[a] Mickaël Beaudhuin,[a] Jean-Charles Dupin,[d] Joachim Allouche,[d] Yannick Guari,[a] Joulia Larionova,[a] Eva Baldrich[b,e*]

[a] *ICGM, University of Montpellier, CNRS, ENSCM, 34000 Montpellier, France.*

[b] *Diagnostic Nanotools Group. Hospital Vall d'Hebron Institut de Recerca (VHIR), Barcelona, Spain.*

[c] *Universitat Autònoma de Barcelona (UAB), Bellaterra, Spain.*

[d] *Institut des Sciences Analytiques et de Physicochimie pour l'Environnement et les Matériaux, UMR 5254, E2S UPPA, CNRS, IPREM, 64000 Pau, France.*

[e] *Centro de Investigación Biomédica en Red de Enfermedades Infecciosas (CIBERINFEC), Instituto de Salud Carlos III, Madrid, Spain.*

List of Abbreviations

**a**	Lattice parameter	**PB**	Prussian blue
**BG**	Berlin green	**PBNPs**	Prussian blue nanoparticles
**C1**	In situ synthesis of PB/PEI NPs	**PB/PEI NPs**	(PEI) mediated PBNPs
**CAT**	Catalase	**PEDOT:PSS**	Poly(3,4-ethylenedioxythiophene)–poly(styrenesulfonate)
**Cb**	Bare PBNPs	**PEI**	Polyethyleneimine
**Cu/Fe HCFs**	Copper-iron hexacyanoferrate	**POD**	Peroxidase
**CV**	Cyclic Voltammetry	**PW**	Prussian white
**EDTA**	Ethylenediaminetetraacetic acid	**PY**	Prussian Yellow
**EDX**	Energy Dispersive X-ray spectroscopy	**ROS**	Reactive oxygen species
**FTIR**	Fourier Transform InfraRed spectroscopy	**SEM**	Scanning Electron Microscopy
**GOx**	Glucose oxidase	**SOD**	Superoxide dismutase
**HCFs**	Hexacyanoferrates	**SPCE**	Screen-printed carbon electrodes
**HRP**	Horseradish peroxidase	**T1, T2, T3**	PB/PEI NPs synthesised by thermally reductive process
**JCPDS**	Joint Committee on Powder Diffraction Standards	**TEM**	Transmission Electron Microscopy
**Km**	Michaelis−Menten constant	**TMB**	3,3',5,5'-Tetramethylbenzidine
**LOD**	Limit of detection	**UV-vis**	Ultraviolet-Visible spectroscopy
**Ms**	Microstrain	**V1**	PB/PEI NPs synthesised by the Vortex process
**Ni/Fe HCFs**	Nickel-iron hexacyanoferrate	**Vmax**	Maximum reaction velocity
**NPs**	Nanoparticles	**XPS**	X-Ray Photoelectron spectroscopy
**PAH**	Poly(allylamine hydrochloride)	**XRD**	X-Ray Diffraction

S1. Schematic illustration of the customized vortex reactor.

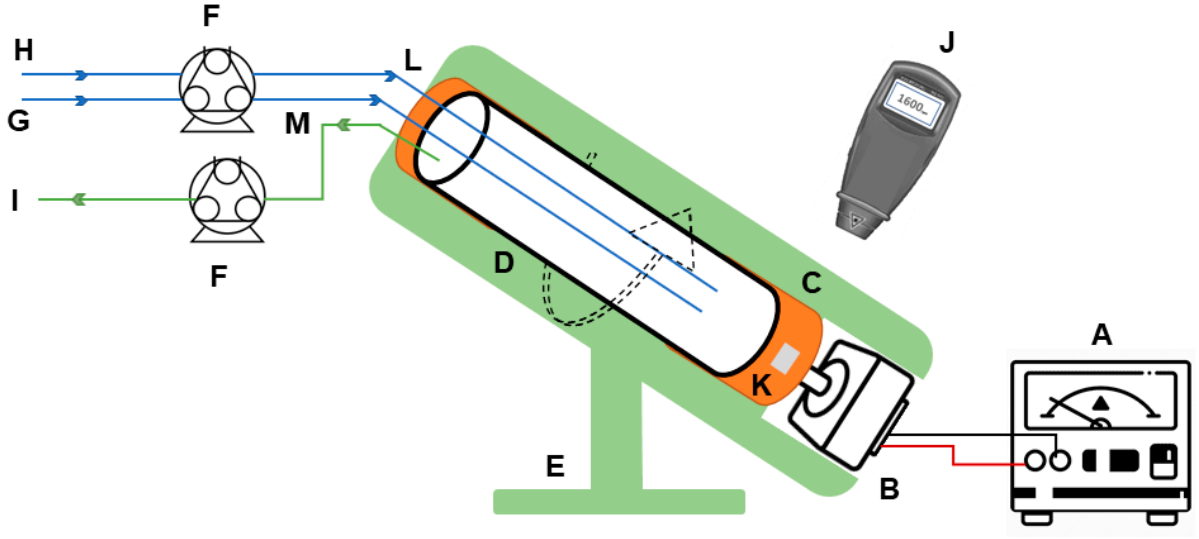

**A**	Laboratory power supply
**B**	DC Motor
**C**	Reactor holder
**D**	Glass reactor
**E**	Customized metal stand
**F**	Peristaltic pump (multichannel)
**G**	Inlet-1 ($FeCl_3.6H_2O$)
**H**	Inlet-2 ($PEI/Na_4Fe(CN)_6.10H_2O$)
**I**	Overflow outlet
**J**	Photo laser tachometer
**K**	Reflective patch
**L**	Metal needle
**M**	Silicon tubing

Scheme S1; Schematic illustration of the vortex reactor.

Table S1; Metal composition and synthesis information of synthesised PBNPs and PB/PEI NPs.

Nanoparticle type		Synthesis Protocol	Fe%	Na%	K%
**Cb**	PBNPs	Co-precipitation	85.06	14.94	
**C1**	PB/PEI NPs	In situ co-precipitation	88.63	11.37	
**V1**	PB/PEI NPs	Vortex	93.98	6.02	
**T1**	PB/PEI NPs	Thermal reduction-refluxed	92.41		7.92
**T2**	PB/PEI NPs	Thermal reduction-refluxed	85.13		14.87
**T3**	PB/PEI NPs	Thermal reduction-room temperature	92.71		7.29

Table S2; Crystallite size, microstrain and lattice parameter of the PBNPs and PB/PEI NPs obtained by pattern matching of DRX pattern and compared with TEM average apparent size.

	DRX				TEM
**Nanoparticle**	**Crystalline size (nm)**	**Average Microstrain (%)**	**a = b = c (Å)**	χ^2	**Apparent average size (nm)**
**Cb**	91	0.09	10.17163	1.607	99±13
**C1**	62	0.08	10.16625	1.713	92±23
**V1**	23	0.9	10.17931	1.19	19±4
**T1**	18	0.66	10.15566	1.19	26±4
**T2**	73	0.18	10.16788	1.82	60±11
**T3**	45	0.08	10.15285	2.24	49±10

Table S3; FTIR peaks analysis.

Vibrational Mode	Cb (cm^{-1})	C1 (cm^{-1})	V1 (cm^{-1})	T1 (cm^{-1})	T2 (cm^{-1})	T3 (cm^{-1})	PEI (cm^{-1})
OH/HOH st.	3210 (b)	3200	3207	3204	3220	3230	
OH/HOH st.	3646 (m)	3635	3633	3634	3635	3632	
OH Cryst. st	3596 (m)	3595	3589	3594	3577	3575	
C≡N st.	2063 (s)	2067	2050	2067	2064	2058	
HOH ben.	1604 (s)	1606	1606	1604	1603	1604	
OH ben.	1421 (s)	1415	1410	1414	1414	1410	
Fe^{2+}-CN st.	598 (s)	601	597	602	601	601	
Fe^{2+}-CN ben.	498 (s)	498	490	490	495	495	
N-H st. (w)		3249	3216	3249	3255	3244	3273
C-H str. (m)							2808<br>2938
N-H ben. (w)		1650	1654	1657	1650	1649	1590
N-H wag. (w)		783	778				760
C-H ben. (w)		1476	1475		1470		1460
C-H ben. (w)		1375	1363	1373	1372	1375	1356
C-N str. (m)		1073	1081				1070

Table S4; Cyclic voltammetry peak analysis.

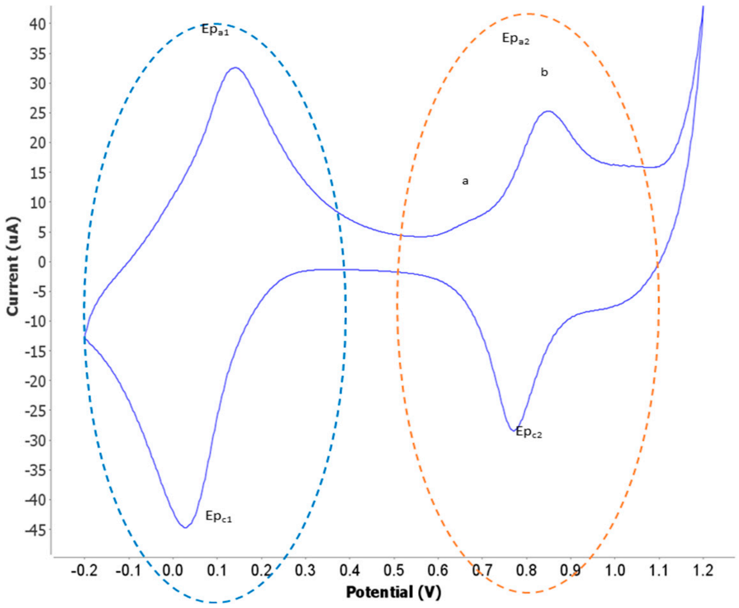

	First Redox Process					Second Redox Process							
	Ox1	Red1	ΔEp1 = Epa1 - Epc1	Ox1	Red1	Ox2		Red2	ΔEp2 = Epa2 - Epc2		Ox2		Red2
Name of PBNPs	Voltammetric Peaks (Potential, V)		Electrochemical Parameters	Voltammetric Peaks (Current, µA)		Voltammetric Peaks (Potential, V)			Electrochemical Parameters		Voltammetric Peaks (Current, µA)		
	Ep_{a1}	Ep_{c1}	ΔEp_1, V	Ip_{a1}	Ip_{c1}	Ep_{a2} a	Ep_{a2} b	Ep_{c2}	ΔEp_2, V a	ΔEp_2, V b	Ip_{a2} a	Ip_{a2} b	Ip_{c2}
**Cb**	0,18	0,04	0,14	21,23	21,00	none	0,86	0,78	none	0,08	none	8,37	14,55
**C1**	0,19	-0,02	0,20	28,20	19,10	none	0,83	0,77	none	0,06	none	12,50	17,17
**T1**	0,15	0,02	0,13	55,50	67,46	0,58	0,84	0,76	-0,18	0,08	0.688	13,37	29,25
**T2**	0,23	-0,05	0,28	13,79	5,65	none	0,75	0,72	none	0,03	none	7.93	8,61
**T3**	0,16	0,22	-0,06	48,36	53,21	0,64	0,86	0,78	-0,13	0,09	0.698	14,76	27,83
**V1**	0,16	0,04	0,12	29,23	21,69	none	0,84	0,77	none	0,07	none	5,40	15,71

Table S5; Catalytic activity of the particles - LOD and Kinetic parameters.

Nanoparticle type	Vmax (10^{-8}M s^{-1})	Km (mM)	Catalytic activity LOD in TMB (ng/mL)
**Cb**	4.29	6.03	27.1
**C1**	4.30	4.10	32.7
**V1**	4.11	4.39	18.3
**T1**	3.38	2.66	32.6
**T2**	4.35	85.19	77
**T3**	4.76	3.35	25.5

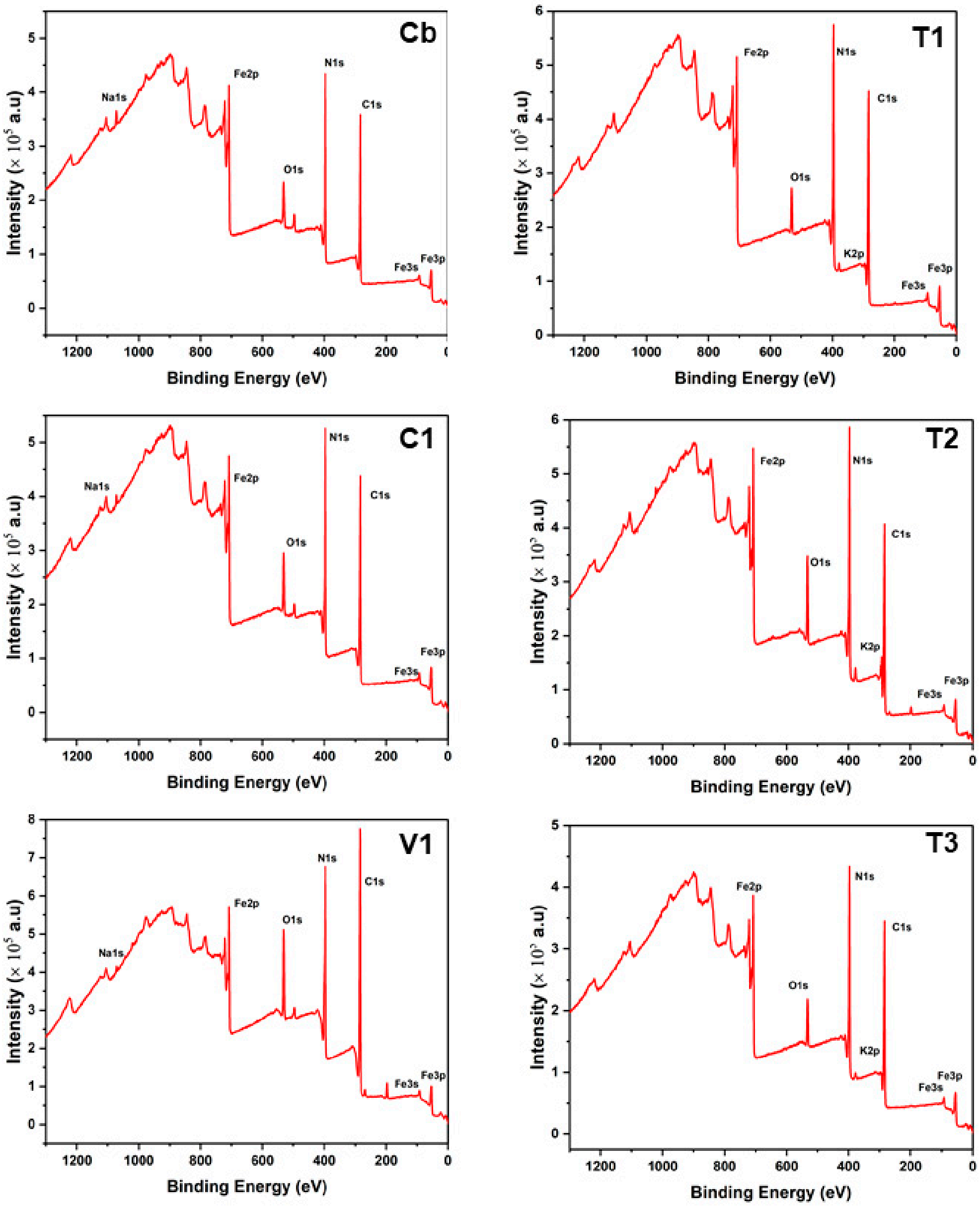

Figure S1; XPS elemental survey spectra for PBNPs and PB/PEI NPs.

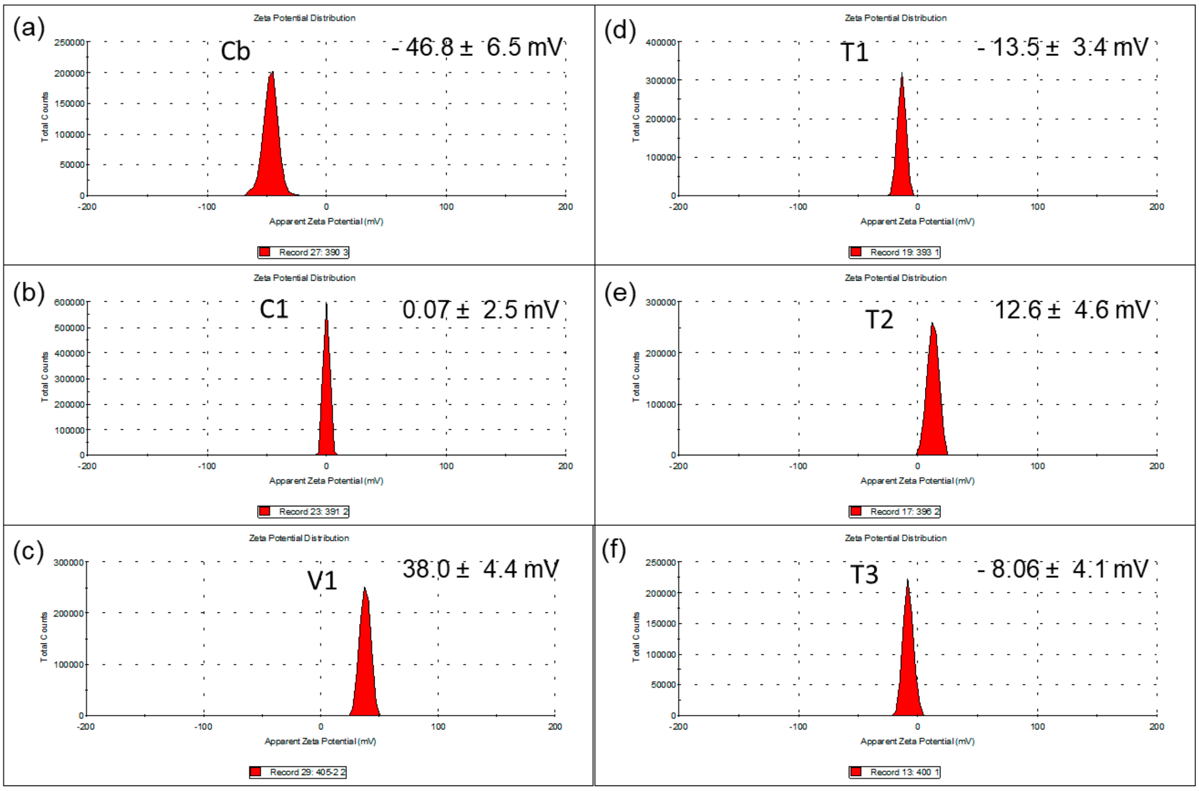

Figure S2; Zeta potential analysis of PBNPs and PB/PEI NPs (a) **Cb**, (b) **C1**, (c) **V1**, (d) **T1**, (e) **T2**, and (f) **T3**.

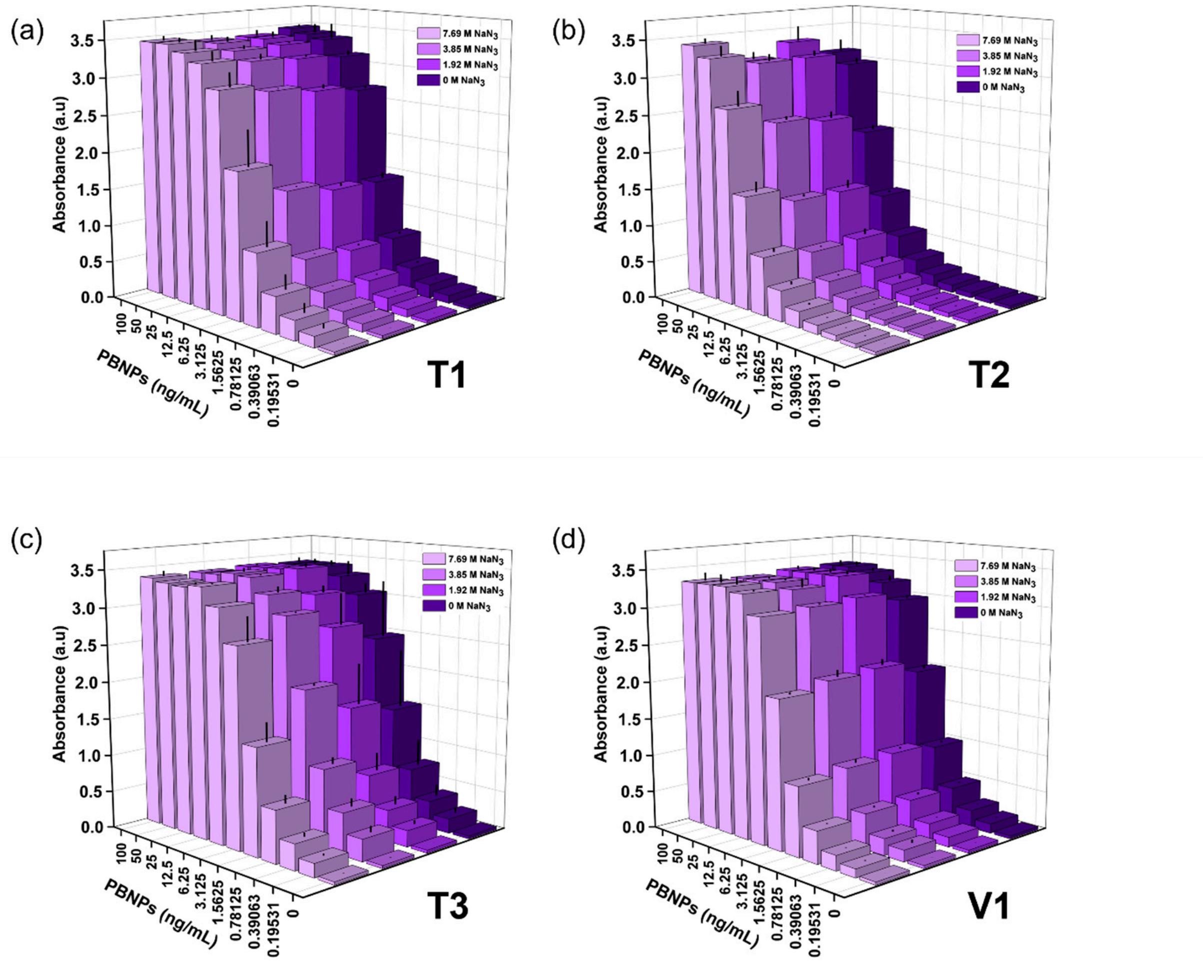

Figure S3; Inhibition in the catalytic activities of the synthesized nanozymatic PB/PEI NPs by NaN_3. POD-like activity of (a) **T1**, (b) **T2**, (c) **T3** and (d) **V1** in the presence of 7.69, 3.85, 1.92 and 0 M of NaN_3.

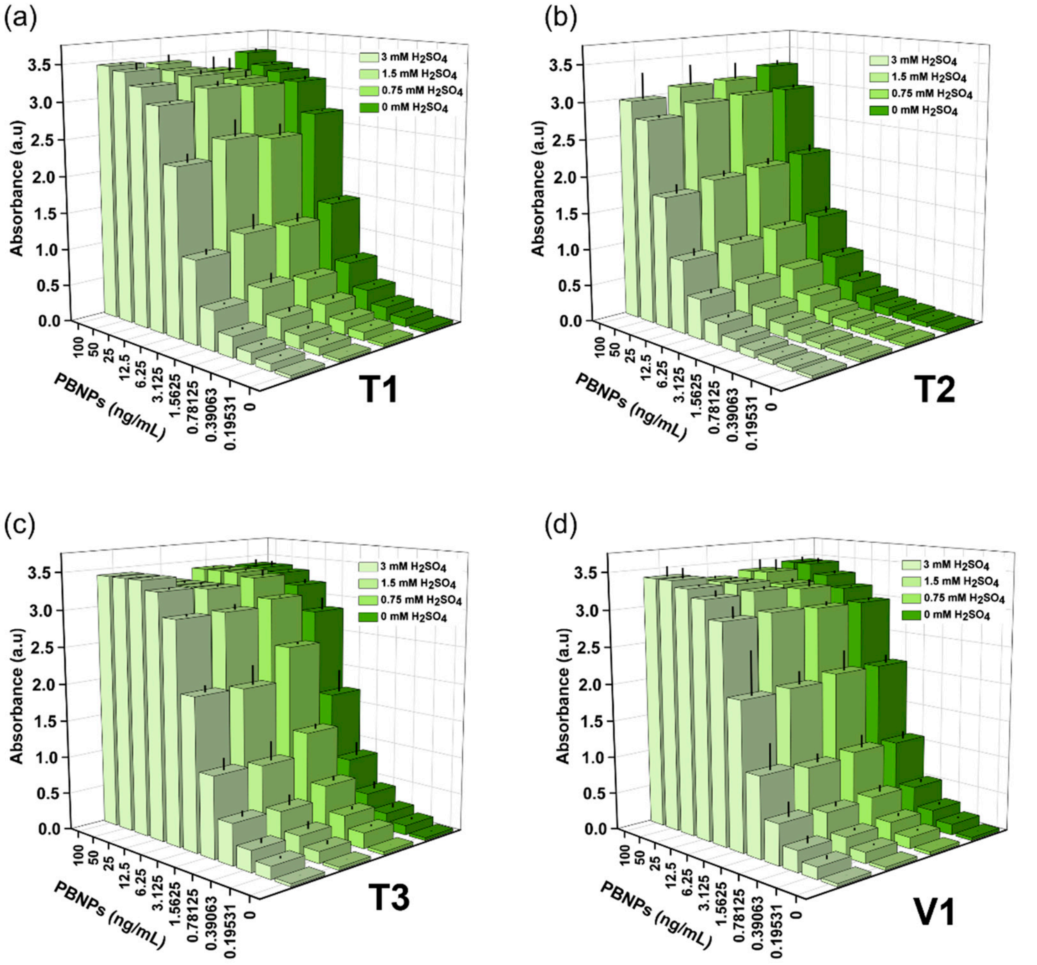

Figure S4; Inhibition of the catalytic activities of the synthesized nanozymatic PB/PEI NPs in the presence of H_2SO_4. POD-like activity of (a) **T1**, (b) **T2**, (c) **T3** and (d) **V1** in the presence of 3, 1.5, 0.75 and 0 M of H_2SO_4.

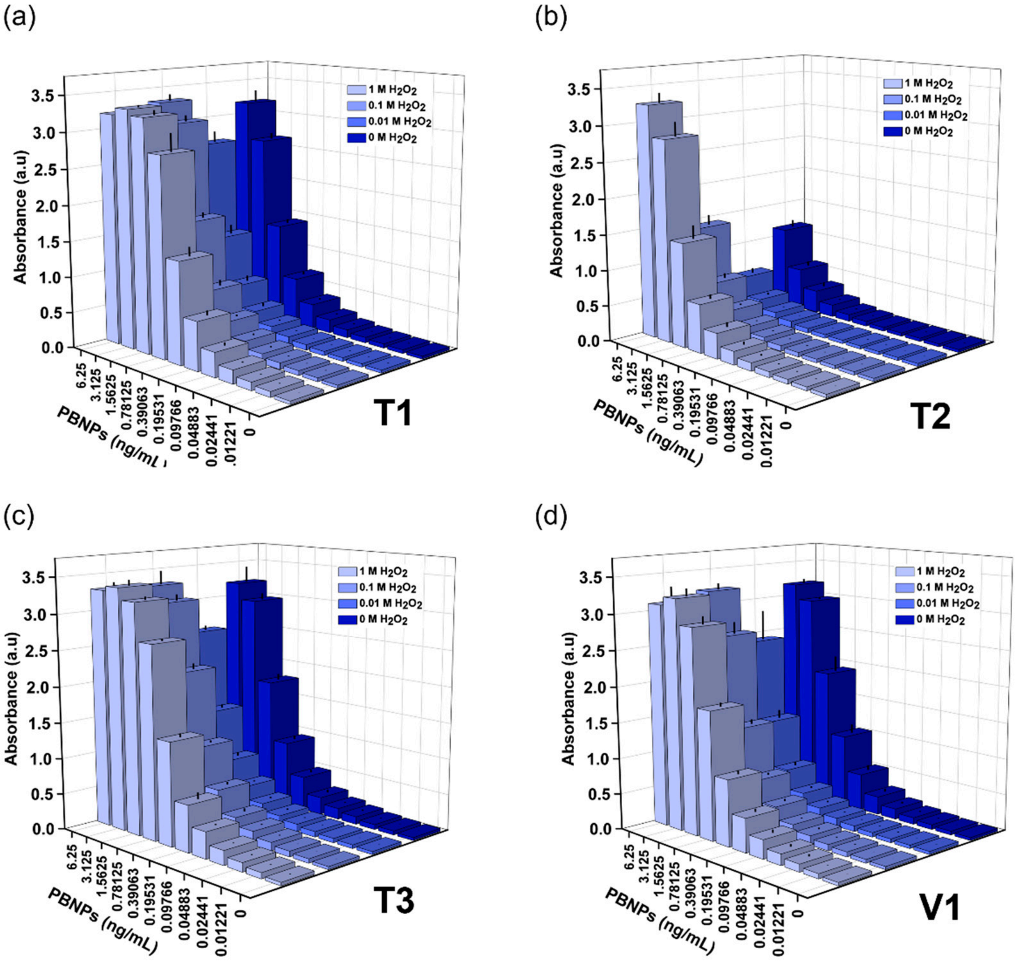

Figure S5; Inhibition of the catalytic activities of the synthesized nanozymatic PB/PEI NPs by high concentrations of H_2O_2. POD-like activity of (a) **T1**, (b) **T2**, (c) **T3** and (d) **V1** in the presence of 1, 0.1, 0.01 and 0 mM of H_2O_2.

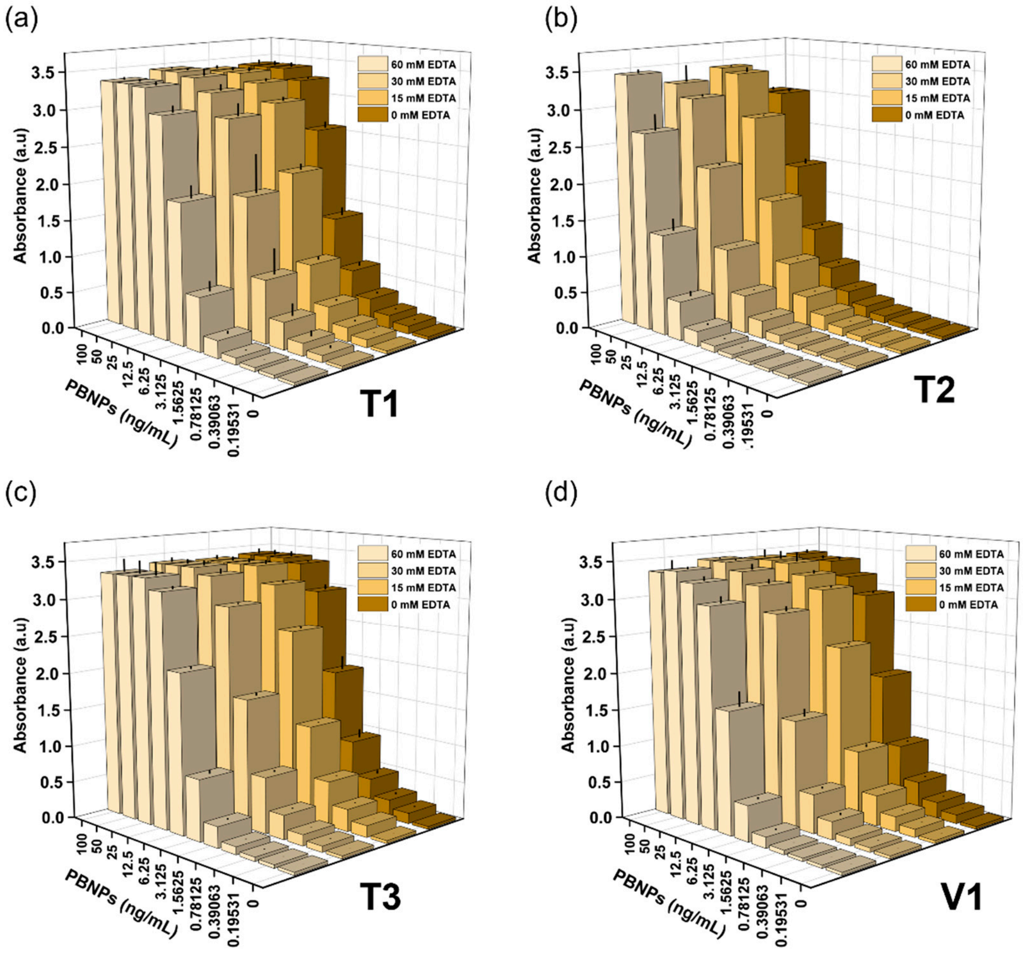

Figure S6; Inhibition of the catalytic activities of the synthesized nanozymatic PB/PEI NPs in the presence of EDTA. POD-like activity of (a) **T1**, (b) **T2**, (c) **T3** and (d) **V1** in the presence of 60, 30, 15, and 0 mM of EDTA.
